# Study of the Location and Morphology of the Pterion in Adult Nigerian Skulls

**DOI:** 10.5402/2013/403937

**Published:** 2013-06-12

**Authors:** Sunday A. Adejuwon, Funmilayo E. Olopade, Modupe Bolaji

**Affiliations:** Department of Anatomy, University of Ibadan, Ibadan, Nigeria

## Abstract

The pterion which marks the union of 4 bones of the cranium is located superior to the zygomatic arch and posterior to the frontozygomatic suture. It is an important neurosurgical landmark for the lateral/pterional approach and has racial differences in both its location and pattern of union of the bones. This study aims to analyze the location and types of pterion in adult Nigerian skulls. Bilateral sides of 37 adult dry skulls were studied. The pterion types were classified; linear distances from the centre of the pterion to the midpoint of the zygomatic arch and to the frontozygomatic suture were measured; these were analyzed for side and gender differences. Sphenoparietal was the most common pterion type (86.1%) followed by frontotemporal (8.3%), stellate (5.6%), and epipteric types (0%). The mean distances from the pterion to the midpoint of zygomatic arch were 39.74 ± 0.505 mm and 37.95 ± 0.657 mm in males and females, respectively, while the distances to the frontozygomatic suture were 31.87 ± 0.642 mm and 30.35 ± 0.836 mm. The vertical position of the pterion was significantly higher in males than females. Bilateral occurrence is statistically insignificant. This information will be of neurosurgical and anthropological importance.

## 1. Introduction 

The pterion is a craniometric point near the sphenoid fontanelle of the skull. It is a point of convergence of the sutures between the frontal, sphenoid, parietal, and squamous temporal bones [1]. There are varied patterns of articulation of these bones and sometimes a small epipteric bone may be present. There are four types of sutural pattern: sphenoparietal, the sphenoid and parietal bones are in direct contact; frontotemporal, the frontal and temporal bones are in direct contact; stellate, all the four bones meet at a point; and epipteric, where there is a small sutural bone uniting all the bones [2].

The pterion is located superior to the zygomatic arch and posterior to the frontozygomatic suture. This area is known as the weakest part of the skull, yet it overlies the course of the anterior division of the middle meningeal artery [1], thus making it vulnerable to rupture, leading to extradural hematoma in the event of a blunt trauma to the side of the head [3]. In addition, it acts as an important landmark for locating the Broca's motor speech area, anterior pole of the insula, and middle cerebral artery [4]. The “pterional” or lateral approach is occasionally used in operations involving the Broca's motor speech area [5] and repairing aneurysms of the middle cerebral artery [6].

Differences in the exact location of the pterion have been observed among different races, and this could be due to genetic or environmental influences affecting the craniometric indices of human skull. This study is thus of immense benefit to neurosurgical procedures on Nigerians skull.

## 2. Materials and Methods

Sixty-two dry human skulls from the Department of Human Anatomy, University of Ibadan, were assembled for this study. The skulls were from prosected specimens of dissected cadavers used for medical students' training. The cadavers were sourced from Lagos, Ile-Ife, Ibadan, and other cities within the southwestern region of Nigeria. Eruption of the third molar was used to identify the adult skulls. Thirty-seven adult skulls were selected for the study after exclusion of those with deformities and trauma affecting the landmarks for measurement, for example, fractures of the zygomatic arch. 

The skulls were divided into 21 males and 16 females using gross dimorphic characteristics for sexual characterization [1]. Inspection of the pterion was carried out and classified into four types: sphenoparietal, frontotemporal, stellate, and epipteric according to previous classifications [2].

**Figure 1:**
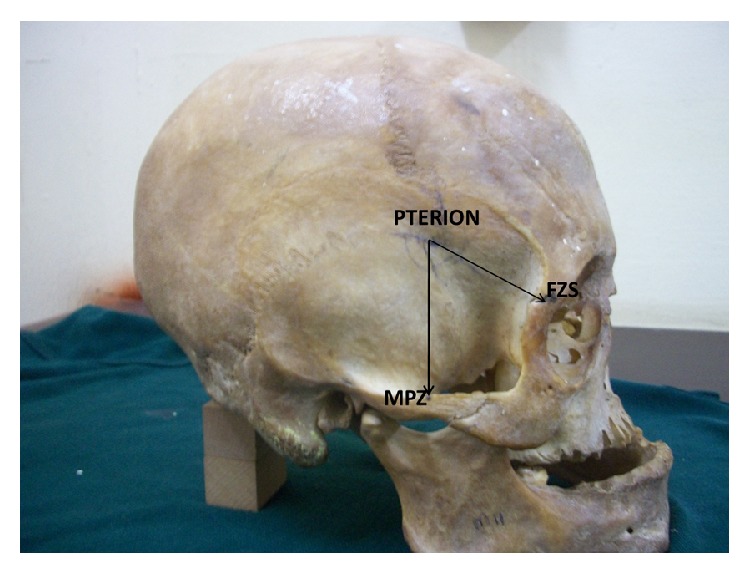
Skull showing measurements taken from the pterion to the midpoint zygomatic arch (MPZ) and to the frontozygomatic suture (FZS).

Measurements (Figure 1) were taken on both sides of the skull from the pterion to the midpoint of zygoma (MPZ) and to the frontozygomatic suture (FZS) using a manual vernier calipers with an accuracy of 0.01 mm. 

Data obtained were analyzed using GraphPad Prism4 software. Means and standard deviations were generated and compared using the Student's *t*-test for the assessment of side and gender differences. *P* value <0.05 was considered significant.

## 3. Results

**Figure 2:**
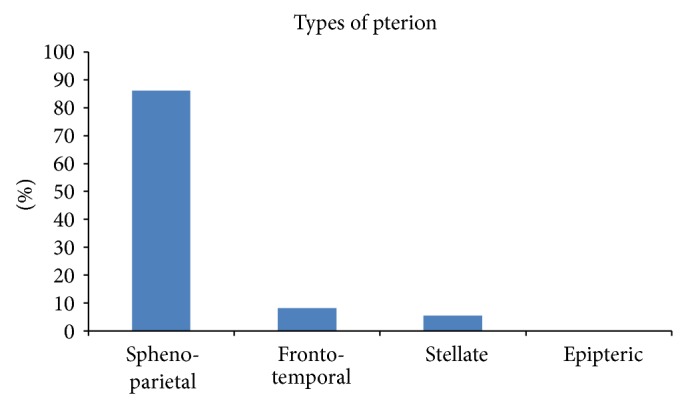
Graph showing the distribution of the types of pterion present in the Nigerian skulls.

**Figure 3:**
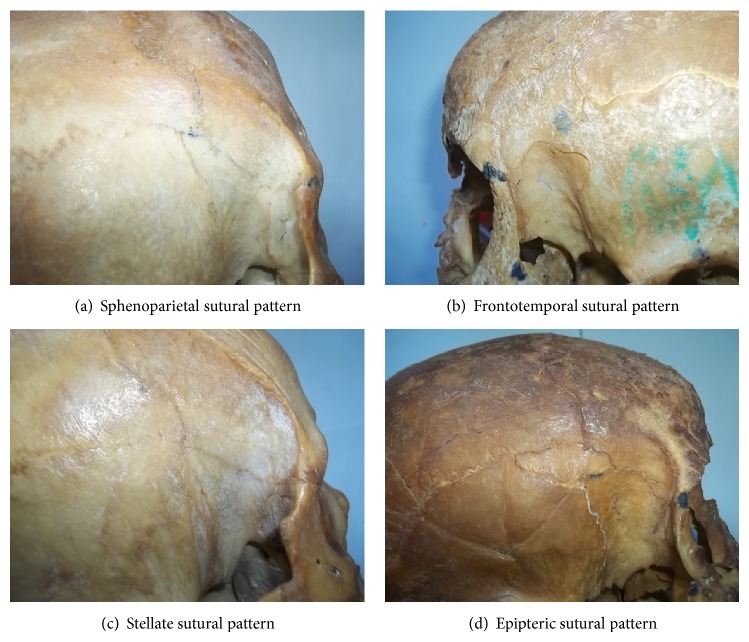
Photographs illustrating the four different types of pterion sutural patterns.

The occurrence of the different types of pterion in the Nigerian skulls is represented in Figure 2.  Figure 3 shows the different types of pterion sutural patterns. The sphenoparietal type was the most common type (86.1%), followed by the frontotemporal (8.3%) and stellate (5.6%) types. There was no epipteric type of suture present among the skulls.

The means and standard deviations of the various measurements taken from the pterion are presented in Table 1. The distance of the pterion posterior to the frontozygomatic suture and superior to the midpoint of the zygomatic arch was compared between the male and female skulls. Comparison between the left and right side of all the skulls was also done.

The study revealed a statistically significantly higher pterion in the male skulls when compared with the females, but no side differences were observed.

## 4. Discussion 

The sphenoparietal type of pterion was observed to be the most common among the Nigerian skulls, just as has been reported in different races, for example, Indians, Turks, and Kenyans [7–9]. However, none of them had the epipteric type of sutural pattern. Though the actual determinants of the formation of the pterion are unknown, articulation of the cranial bones is thought to be under genetic influence especially the MSX2 gene [10]. Ethnic and racial variations are thus commonly observed. The fact that the development of the calvarium is tightly coordinated with the growth of the brain may explain the prevalence of frontotemporal pattern of sutures among monkey skulls as reported by Wang et al. [11] unlike humans with larger brains who have a predominantly sphenoparietal pattern of suture.

The pterion was located 39.74 ± 0.505 mm and 37.95 ± 0.657 mm above the midpoint of the zygomatic archin males and females, respectively. This is higher than that observed in Koreans reported as 36.9 ± 3.8 mm [12] and Indians reported as 38.5 mm [7]. However, it is similar to the 39.31 ± 3.28 mm and 37.35 ± 2.97 mm in males and females, respectively, reported in a Kenyan study [9]. This is likely due to the slightly higher arch of the cranium in Africans when compared to the Asians.

The pterion has been widely reported to be higher in males than in females [7, 9], and this study reports a similar finding. Reasons proffered for this include the observations from morphometric studies that female skulls are shorter and broader in proportion than male ones [1] and the presence of a more robust zygoma in males [13].

The pterion was located 31.87 ± 0.642 mm in males and 30.35 ± 0.8358 mm in females posterior to the frontozygomatic suture. This concurs with description by Williams et al. [1] that the pterion lies 30 to 35 mm away from the frontozygomatic suture and similar to the Kenyan study which was reported as 30.35 ± 3.61 mm posterior to the frontozygomatic suture. However, there are wide variations with reports of 26.8 ± 4.5 mm [12] and 35 ± 5 mm [14] in Koreans and Turks, respectively.

There were no significant side variations in the distance from the midpoint of pterion to the frontozygomatic suture and midpoint of zygoma among all the skulls; hence the landmarks can be used to locate the pterion irrespective of the side. This information is of great importance for neurosurgical operations in regions where neuronavigation equipments are unavailable and for anthropological identification of Nigerian skulls.

## Figures and Tables

**Table 1:** Comparison of the measurement of the distance of the pterion from the midpoint of zygomatic arch (MPZ) and the frontozygomatic suture (FZS) between sexes and sides.

Measurements	Males (mm)	Females (mm)	*P* value
From pterion to MPZ	39.74 ± 0.505	37.95 ± 0.657	0.032
From pterion to FZS	31.87 ± 0.642	30.35 ± 0.8358	0.146

Measurements	Right side (mm)	Left side (mm)	*P* value

From pterion to MPZ	39.1 ± 0.583	38.77 ± 0.631	0.704
From pterion to FZS	31.52 ± 0.677	30.82 ± 0.809	0.505

## References

[1] Williams L., Bannister L., Berry M., Collins P., Dyson M., Dussek E. (1998). *Gray’s Anatomy*.

[2] Murphy T. (1956). The pterion in the Australian aborigine. *The American Journal of Physical Anthropology*.

[3] Lama M., Mottolese C., Alvisi C., Riccio A. (2000). Middle meningeal artery aneurysm associated with meningioma. *Journal of Neurosurgical Sciences*.

[4] Apinhasmit W., Chompoopong S., Chaisuksunt V., Thiraphatthanavong P., Phasukdee N. (2011). Anatomical consideration of pterion and its related references in thai dry skulls for pterional surgical approach. *Journal of the Medical Association of Thailand*.

[5] Lindsay K., Bone I., Callander R. (1991). *Neurology and Neurosurgery Illustrated*.

[6] Escosa-Bagé M., Sola R. G., Liberal-González R., Caniego J. L., Castrillo-Cazón C. (2002). Fusiform aneurysm of the middle cerebral artery. *Revista de Neurologia*.

[7] Saxena S. K., Jain S. P., Chowdhary D. S. (1988). A comparative study of pterion formation and its variations in the skulls of Nigerians and Indians. *Anthropologischer Anzeiger*.

[8] Oguz Ö., Şanli S. G., Bozkir M. G., Soames R. W. (2004). The pterion in Turkish male skulls. *Surgical and Radiologic Anatomy*.

[9] Mwachaka P., Hassanali J., Odula P. (2008). Anatomic position of the pterion among Kenyans for lateral skull approaches. *International Journal of Morphology*.

[10] Hussain Saheb S., Mavishetter G. F., Thomas S. T., Prasanna L. C., Magi M. P. (2011). A study of sutural morphology of the pterion and asterion among human adult Indian skulls. *Biomedical Research*.

[11] Wang Q., Opperman L. A., Havill L. M., Carlson D. S., Dechow P. C. (2006). Inheritance of sutural pattern at the pterion in rhesus monkey skulls. *Anatomical Record A*.

[12] Lee U., Park D., Kwon S., Paik D., Han S. (2001). Morphological analysis of pterion in Korean. *Korean Journal of Physical Anthropology*.

[13] Ikeda T., Nakamura M., Itoh M. (1999). Sex differences in the zygomatic angle in Japanese patients analyzed by MRI with reference to moire fringe patterns. *Aesthetic Plastic Surgery*.

[14] Ilknur A., Mustafa K. I., Sinan B. (2009). A comparative study of variation of the pterion of human skulls from 13th and 20th century anatolia. *International Journal of Morphology*.

